# Development of high-titer class-switched antibody responses to phosphorylated amino acids is prevalent in pancreatic ductal adenocarcinoma

**DOI:** 10.3389/fimmu.2025.1501943

**Published:** 2025-03-28

**Authors:** Talita Aguiar, Shunya Mashiko, Kesava Asam, Poulomi Roy, Shikun Wang, Katharina Frank, Max Dietzel, Luca G. Z. Schahadat, Mattea Ausmeier, Andrea Hertel, Zhe Ran Susan Duan, Bradley Aouizerat, Jeanine M. Genkinger, Helen Remotti, Emmanuel Zorn

**Affiliations:** ^1^ Columbia Center for Translational Immunology, Columbia University Irving Medical Center, New York, NY, United States; ^2^ Dental Translational Research Center, New York University, New York, NY, United States; ^3^ Department of Biostatistics, Columbia University Mailman School of Public Health, New York, NY, United States; ^4^ Medical Department IV - Großhadern, LMU University Hospital, Ludwig-Maximilians-Universität (LMU) München, Munich, Germany; ^5^ Department of Pathology and Cell Biology, Columbia University Irving Medical Center, New York, NY, United States; ^6^ Department of Epidemiology, Columbia University Mailman School of Public Health, New York, NY, United States; ^7^ Herbert Irving Comprehensive Cancer Center, Columbia University Irving Medical Center, New York, NY, United States

**Keywords:** pancreatic ductal adenocarcinoma, antibody responses, phosphoryl adducts, phosphoproteome, ELISA

## Abstract

While immunotherapy tends to be ineffective against pancreatic ductal adenocarcinoma (PDAC), this cancer type often elicits B-cell immunity. However, the exact antigens responsible for these spontaneous immune responses are still unclear. This study used a unique high-dimensional ELISA to analyze IgG responses to 93 post-translational modifications and other chemical determinants in PDAC patients at the time of diagnosis and before therapy. Results identified 13 specific targets of serum IgG that distinguished PDAC patients from healthy donors. Phosphorylated-serine, -threonine, and -tyrosine emerged as the primary targets, with most patients showing high-titer IgG, predominantly of the IgG1 and IgG3 subclasses. Moreover, serum reactivity to these phosphorylated residues was higher in patients with metastatic disease, suggesting a relation between B cell immunity and tumor burden. Lastly, immunofluorescence staining and phosphoproteomic analysis provided evidence of the accumulation of phosphorylated amino acids in PDAC cells and identified a series of consensus abnormal phosphosites. Overall, our findings reveal for the first time the development of robust antibody responses targeting phosphorylated residues in PDAC.

## Introduction

The incidence of pancreatic cancer, especially its most frequent form pancreatic ductal adenocarcinoma (PDAC), has markedly increased over the past several decades and now ranks as the fourth leading cause of cancer death in the United States (1). The estimated new cases and deaths from pancreatic cancer in the United States in 2024 were 66,440 and 51,750 respectively (1). Only 8.5% of PDAC patients live beyond 5 years after their initial diagnosis. This dismal survival rate is largely explained by the fact that most pancreatic cancer cases are detected at a late stage when treatments have minimal efficacy. Currently, blood levels of sialyl Lewis A, a.k.a. carbohydrate antigen 19.9 (CA19.9), is the sole FDA-approved marker for PDAC. Unfortunately, this marker is only useful in symptomatic patients but has very low predictive value (0.5-0.9%) as a screening marker in asymptomatic individuals (2). Effective early detection markers are therefore crucially needed for this deadly cancer.

Naturally occurring antibody responses to tumors are well-documented across various human cancers (3, 4). These responses often emerge in the early stages of tumor growth, even before other signs of cancer become apparent. Early studies using the SEREX (serological analysis of recombinant tumor cDNA expression libraries) method identified two main types of tumor antigens targeted by humoral immunity: 1) “cancer testis antigens” corresponding to proteins abnormally expressed in cancer cells such as MAGE-1 and 2) self-antigens, by far the most abundant, including numerous intracellular proteins (4, 5). The surprising finding that isotype-switched anti-tumor serological responses primarily target self-antigens prompted investigations into their value as early detection markers for multiple neoplasms such as prostate, breast, and lung cancers (6–11). Autoantibodies were also observed in patients with PDAC using protein microarrays or mass spectrometry (12–15). For a minority of autoantigens, overexpression in transformed cells provided a rationale for their immunogenicity. For other targets, the mechanism is less clear.

Cancer cells accumulate distinctive chemical adducts as a result of exposure to toxic chemicals or detrimental environmental factors. Some adducts arise from direct interactions with exogenous electrophilic compounds, while others result from the cellular response to hazardous exposure (16, 17). Anomalous protein post-translational modifications (PTMs), including irregular methylation, acetylation, phosphorylation, SUMOylation or ubiquitination are among modifications observed in malignant cells including that of PDAC (16, 18–20). With respect to their immunogenicity, modified proteins and chemical adducts have long been recognized as potent targets of antibody responses in the context of autoimmunity or other pathological situations (21). Along the same lines, we hypothesized that PDAC elicit robust antibody responses to specific adducts accumulating in the transformed cells. We reason that these responses lead to antibody signatures mirroring the adduct profiles of the tumor cells. Here we used a unique ELISA platform to characterize the anti-adduct reactivity profile associated with PDAC.

## Materials and methods

### Human subjects and specimens

Plasma specimens collected from 50 to 90-year-old subjects with PDAC (N=39) obtained from the Herbert Irving Comprehensive Cancer Center Database Shared Resource (DBSR) and from 50 to 94-year-old control individuals without cancer purchased from Precision for Medicine (Norton, MA, USA), were used in this study. The mean age for both cohorts was 68 years old; 70% were male; 35% had metastasis at diagnosis. We collected 30 plasma samples from patients with type 2 Diabetes (DM2) and 12 plasma samples from patients with advanced PDAC (PDAC-PT) to be tested as a control cohort. For the DM2 patients, we requested blind samples with no information regarding age or sex; the only inclusion criterion was an HbA1c level above 7%. For the PDAC-PT the mean age was 71 years old, and the samples were collected from patients with advanced disease ~55 days after initial treatment. All samples were de-identified according to the Protected Health Information regulations. The study was approved by the Columbia University Institutional Review Board. Subject information is listed in **Supplementary Tables S1A–C**.

### ELISA for the detection of adduct-reactive IgG

IgG reactivity to 93 chemical adducts was assessed using a modified version of a previously described ELISA platform (22). The ELISA panel includes: (i) forty-six synthetic 5-mer peptides, each composed of a modified amino acid flanked by four unmodified arginine, two on each side (5-mer peptides with matching unmodified residues were included in the panel as controls); (ii) 6 modified amino acids; (iii) 8 PTM compounds (e.g., small ubiquitin related modifier (SUMO) and ubiquitin); and (iv) 33 cofactors, coenzymes, and metabolites (e.g., coenzyme Q10). The panel also includes MDA–bovine serum albumin (BSA), insulin, and LPS as controls. MDA-modified BSA and MDA-lysine/arginine peptides were generated by incubating acid-hydrolyzed 1,1,3,3-tetramethoxypropane (Sigma-Aldrich, St. Louis, MO, USA) with BSA or lysine/arginine peptides. Briefly, 2 M 1,1,3,3-tetramethoxypropane was hydrolyzed in 96mMHCl for 15 min at 37°C and then neutralized with NaOH. BSA (2 mg/ml) or lysine/arginine peptides (10 mg/ml) were incubated with 0.2 M MDA for 3 hours at 37°C. Extensive dialysis against 1× phosphate-buffered saline (PBS) was performed at 4°C for 36 hours. All adducts and controls included in the panel are detailed in **Supplementary Table S2** together with their sources and working concentrations.

IgG reactivity to the 93 adducts included in the panel was quantified by ELISA as follows: Corning™ Clear Polystyrene 96-Well EIA/RIA Microplates (Corning Incorporated) were coated in duplicate with adducts, compounds, and control antigens at 4°C for 20 hours. Synthetic peptides with modified amino acids, large PTM compounds, and small compounds were coated at 10 μM, 1 μM, and 1 mM, respectively (**Supplementary Table S2**). ELISA plates were washed three times in PBS Tween (PBST) and then blocked with 3% BSA (Fisher Scientific Inc.) in PBST for 2 hours at 37°C. Plasma diluted in PBST at 1:200 for IgG was distributed to the plate and incubated for 3 hours at room temperature. Plates were then washed five times in PBST and incubated with horseradish peroxidase (HRP)–conjugated anti-human IgG affinity-purified F(ab′) _2_ fragment - Host Donkey IgG; Polyclonal; Dilution 1:4000 - (Jackson ImmunoResearch Laboratories Inc., West Grove, PA) diluted in blocking buffer for 1 hour. Plates were then washed again, and HRP activity was developed with 3,3′,5,5′-tetramethylbenzidine (TMB; Fisher Scientific Inc.). Reactivity to adducts was calculated and reported as an optimal density (OD). To determine the subclasses of plasma IgG in PDAC patients, we used anti-IgG1, anti-IgG2, anti-IgG3, or anti-IgG4 as secondary antibodies (Clone 4E3, HP6002, HP6050 and HP6025 respectively; Southern Biotech, Birmingham, AL - Dilution 1:4000).

### Detection of phosphoryl tyrosine by immunoflurescence

Immunofluorescence staining was performed to detect phosphoryl-tyrosine in tissue using the antibody P-Tyr-1000 (Cell signaling- Host Rabbit mAb mix, dilution 1:100). Thirteen out of the 39 PDAC cases were evaluated including 11 tumor tissue paired with adjacent non-tumoral tissue; 2 tumor tissue without adjacent non-tumoral tissue were also available. Additionally, 4 pancreatic tissue specimens without cancer and 1 tonsil specimen were used as controls. In brief, formalin-fixed paraffin-embedded (FFPE) tissue blocks were deparaffinized with xylene and rehydrated in sequential steps with solutions containing decreasing concentrations of ethanol (100%, 95% and 70%). A DIVA Decloaker (BIOCARE MEDICAL) was used to perform antigen retrieval and slides were then blocked with 3% BSA (Fisher Scientific Inc.) for 1 hour at room temperature. Slides were subsequently incubated overnight at 4°C with anti-P-Tyr mab, followed by 3 washes with PBS 1x. Slides were then incubated for 1 hour at room temperature with a secondary anti-rabbit IgG (H+L), F(ab’)2 fragment antibody conjugated to Alexa Fluor^®^ 488, followed again by 3 washes with PBS 1x Lastly, slides were mounted with Vectashield Vibrance^®^ Antifade Mounting Medium with DAPI (H-1800) (Vector laboratories). Images were acquired with a Zeiss LSM 900 of microscope.

### Liquid chromatography/mass spectrometry-based phosphoproteomics

Flash-frozen tumor specimens and paired adjacent non-tumoral tissue from 3 PDAC cases were used to analyze the phosphoproteome by liquid chromatography/mass sprectrometry (LC-MS/MS) using Creative Proteomics services (New York, USA). Samples were first removed from -80°C and homogenized in 1 mL lysis buffer (8 M urea, 100 mM tris-HCl, pH 8.0, 1% protease inhibitor, 1% phosphatase inhibitor) by beads grinder. All cell debris were removed by centrifugation at 20,000g for 15 min. The protein concentration was then determined using a BCA assay. Approximately 1 mg protein was used for each sample. Disulfide bridges were reduced by 10 mM TCEP at 56°C for 1 h. Reduced cysteine residues were then alkylated by 20 mM iodoacetamide (IAA) in the dark at room temperature for 30 min. The solution was then subject to overnight digestion at 37°C with trypsin (Promega) using an enzyme to substrate ratio of 1:50 (w/w). After digesting, TFA was added to 1% final concentration. Any precipitate was removed by centrifugation at 1,780g for 15 min. Peptide purification was then performed at room temperature on C18 reversed-phase columns. The columns were first conditioned by 100% ACN followed by 0.1% TFA, 80% ACN and then equalized by 0.1% TFA. The acidified and cleared digest were then loaded onto column. After washing by 0.1% TFA, the peptides were eluted from the column by 0.1%TFA, 80% ACN. 10% of the eluent was used as a total proteome sample. The remaining 90% eluent was then processed to phosphopeptide enrichment using Fe-IMAC beads according to manufacturer’s protocol. Briefly, the beads were first resuspended and washed in washing buffer 3 times, and then incubated with peptide solution on a rotator for 30 min. After incubation, the beads were washed 3 times with washing buffer, and 50 μL elution buffer was added to elute the phospho-peptides from the beads. Forty μL 20% TFA was added into the eluate to acidify the eluate. The phospho-peptides were purified again and proceeded to nano LC-MS/MS analysis using an Ultimate 3000 nano UHPLC system (ThermoFisher Scientific, USA), trapping nanocolumn (PepMap C18, 100Å, 100 μm × 2 cm, 5 μm) and an analytical column (PepMap C18, 100Å, 75 μm × 50 cm, 2 μm). The full scan was performed between 300-1,650 m/z at the resolution 60,000 at 200 m/z, the automatic gain control target for the full scan was set to 3e6. The MS/MS scan was operated in Top 20 mode using the following settings: resolution 15,000 at 200 m/z; automatic gain control target 1e5; normalized collision energy at 28%.

### Phosphoproteome differential analysis

Raw MS files were analyzed and searched against *Homo sapiens* protein database (UP000005640) using Maxquant (version 2.3.0.0). The parameters were set as follows: the protein modifications were carbamidomethylating (C), oxidation (M), Phospho (STY); the enzyme specificity was set to trypsin; the maximum missed cleavages were set to 2; the precursor ion mass tolerance was set to 10 ppm, and MS/MS tolerance was 0.5 Da. The analysis was conducted on paired control and tumor samples from 3 patients with PDAC. Differential Phosphorylation was computed using the msqrob2 package: raw intensities were loaded. Peptides with zero intensities were converted to NA. The raw intensities were then log2 normalized and a density plot was generated. Peptides that were only identified in one sample were dropped. The filtered data was then subjected to quantile normalization. A paired differential analysis was performed on the quantile normalized values using Limma package. Cutoff values of 0.05 for the p value and log fold-change < -0.5 or > 1 were applied for selection of a robust list of the phosphorylated differential sites. A heatmap was used to depict protein sites with excess phosphorylation in tumor cells. The quantile normalized values were scaled to make the heatmap. R stats (R version 4.3.3) was used to perform all the analyses. Packages used and reference are reported in **Supplementary Table S3**. The genes corresponding to the phosphorylated proteins were annotated with EntrezIDs using Biomartr package. Pathway analysis was performed using the EntrezIDs and the clusterProfiler package to identify significantly enriched GO terms. Note: We didnt use the FDR p value. We just used the un-adjusted P value. A significance threshold (p < 0.05) was applied, and multiple testing correction methods were employed to control the false discovery rate. Enriched GO terms for biological processes, cellular components and molecular functions were visualized using the R packages reported in **Supplementary Table S3**.

### Statistical analysis

For feature selection, the standardized ELISA readings were subjected to random forest classification using the Boruta package. Confirmed features were then subjected to dimensionality reduction using principal component analysis from tidymodels’ step_pca function. The first three principal components were then visualized using plotly. All the packages used are reported in **Supplementary Table S3**. Unpaired t test with Welch’s correction, Pearson’s correlation coefficients, violin plot and heatmaps were generated using GraphPad Prism 10.

## Results

### Distinctive anti-adduct serum IgG profiles in PDAC

**Figure 1:**
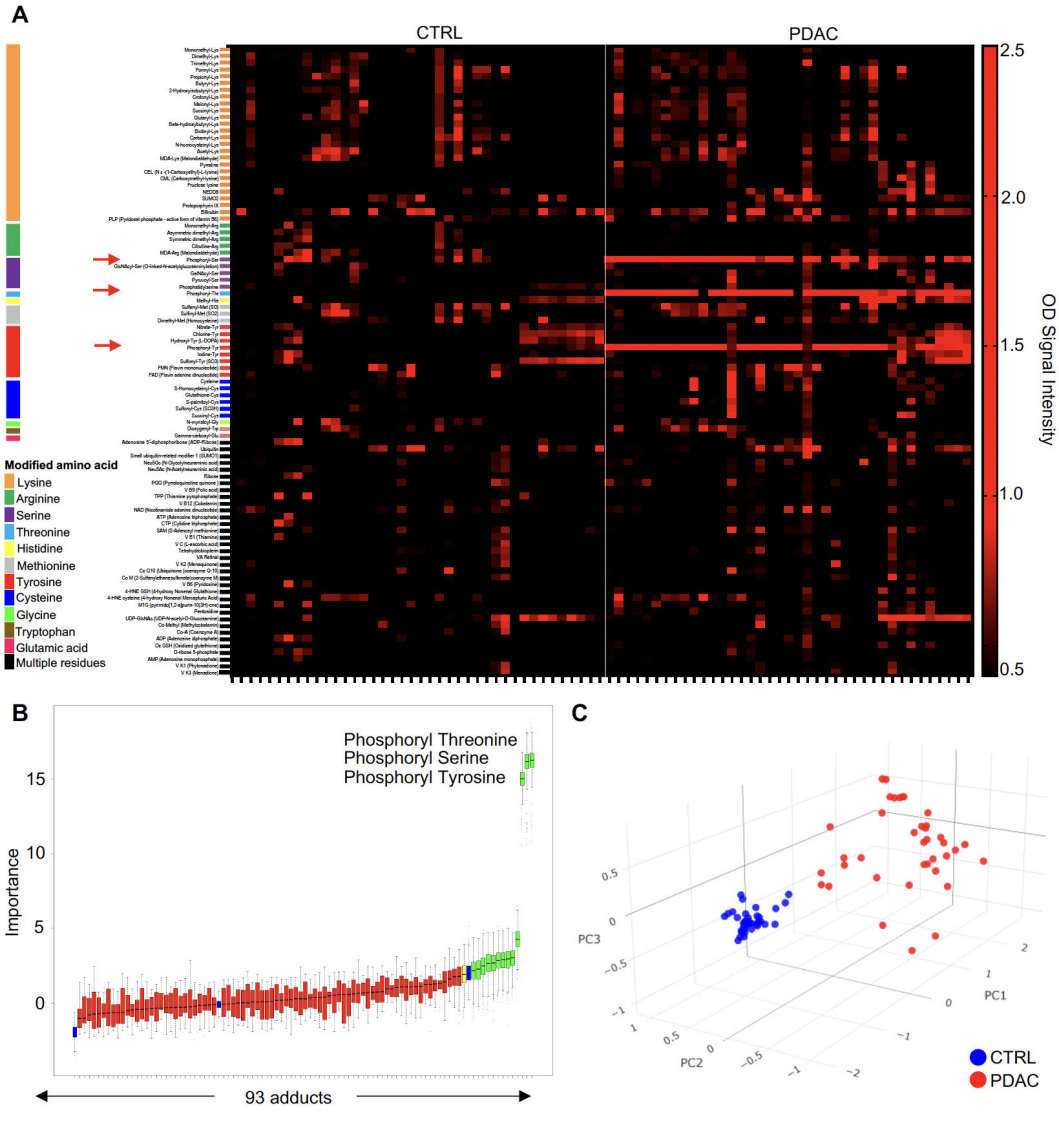
The anti-adduct IgG repertoire in PDAC. **(A)** Heatmap representation of normalized IgG reactivity to 93 adducts in PDAC patients (n=39) and CTRL (n=40); **(B)** Boruta algorithm identified a set of 13 target adducts recognized by IgG that most efficiently discriminated between CTRL and PDAC; **(C)**. PCA of reactivity to the 3 phosphorylated amino acids between PDAC patients (red) and CTRL (blue).

We used an ELISA platform developed in our laboratory to assess IgG reactive to 93 common chemical adducts in the blood of patients with PDAC (N=39; age range 50-90) as well as control individuals without cancer matched for sex and age with cancer cases (N=40; age range 50-94). Importantly, all patient specimens were collected before treatment with chemotherapy. Our adduct panel includes post-translational modifications on 11 amino acids, oxidation-related modifications (e.g., malondialdehyde, MDA), advanced glycation end products (e.g., pyrraline, pentosidine) and certain co-enzymes that qualify as adducts based on their binding properties (e.g., flavin adenine dinucleotide, FAD). **Figure 1A** depicts the reactivity profiles toward all 93 adducts for PDAC cases and HD. As clearly apparent in this figure, reactivity to phosphoryl-threonine (Thr), -serine (Ser) and -tyrosine (Tyr) was markedly increased in cancer patients when compared to HD (red arrows). A random forest classifier built with the Boruta algorithm identified 13 adduct recognized by IgG that most efficiently discriminated between cases and controls (**Figure 1B**; **Supplementary Figure S1**). Phosphoryl-Thr, -Ser and -Tyr were the most significant among the 13 adducts. Spatial distribution based solely on reactivity to these 3 phosphorylated residues revealed two separate clusters corresponding to PDAC cases and HD (**Figure 1C** **).** In contrast a principal component analysis (PCA) based on reactivity to all 93 adducts did not distinguish PDAC cases from CTRL (**Supplementary Figure S2**).

### High-titer class-switched IgG responses to phosphoryl adducts in PDAC

**Figure 2:**
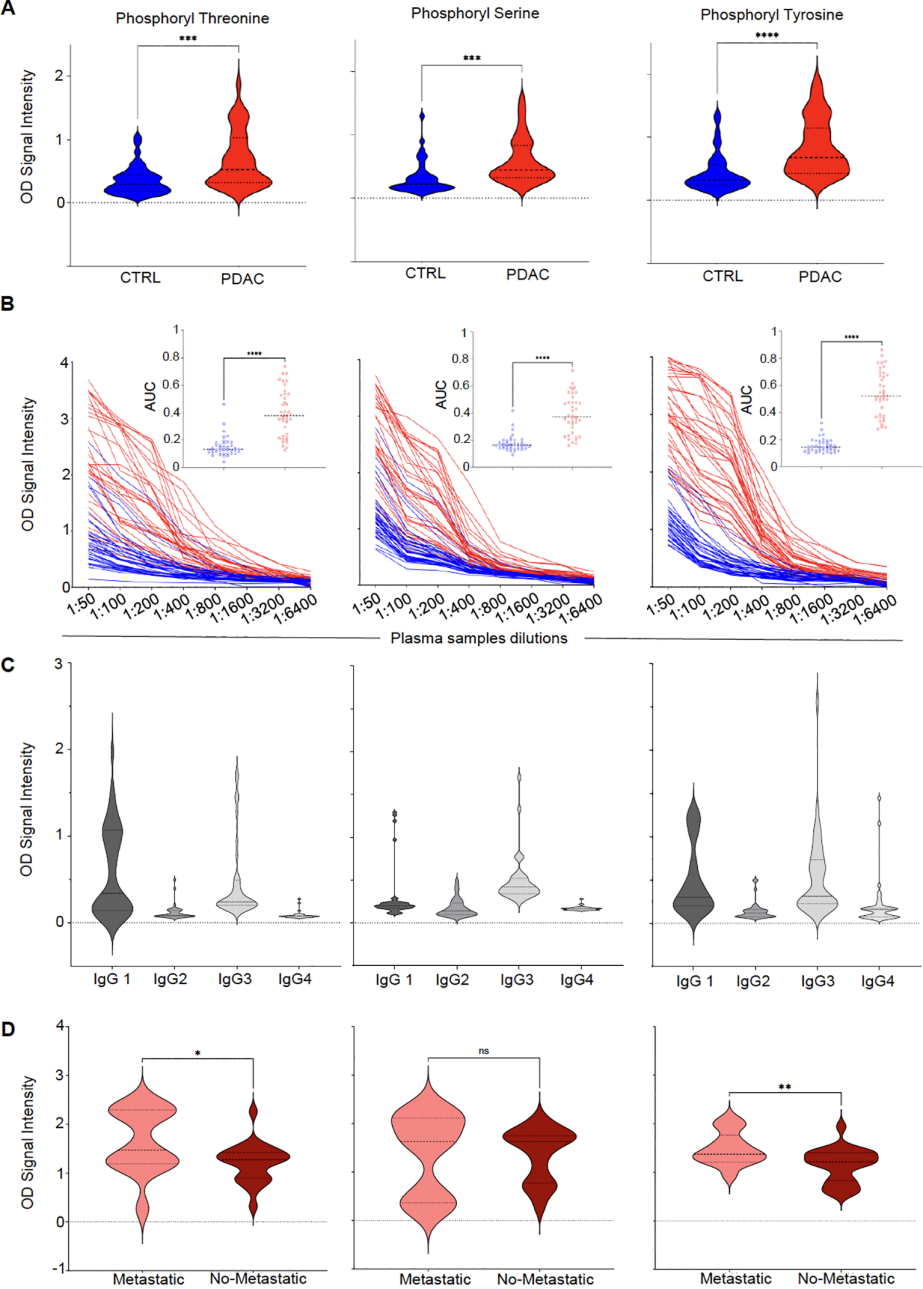
Class-switched high-titer serological responses to phosphoryl adducts in PDAC. **(A)** Differential IgG reactivity to the 3 phosphorylated amino acids between PDAC (red) and controls (CTRL, blue). ****p-value < 0.0001; ***p-value < 0.001 (Unpaired t test with Welch’s correction). **(B)** Titration of IgG reactivity to the 3 phosphoryl adducts in in PDAC patients (red lines, N=39) and controls (CTRL, blue lines, N=40). Insets: Comparison between area under the curves (AUC) calculated for CTRL and PDAC titration curves. ****p<0.00001. **(C)** Subclass determination of IgG reactive to phosphoryl adducts in PDAC using secondary antibodies specific to IgG1–IgG4. **(D)** Differences in IgG reactivity between patients with (n=14 – light red) or without metastasis (n=22 – dark red). *p-value < 0.05; **p-value <0.01 (Unpaired t test with Welch’s correction). ns, No significant.

Differential serum IgG reactivity to the 3 phosphoryl adducts was next validated by ELISA using 5-mer peptides with modified amino acids as baits and unmodified amino acids as controls. As reported in **Figure 2A**, serum reactivity to all 3 targets was significantly elevated in PDAC cases compared to HD. Serum titration confirmed higher reactivity for PDAC patients compared to controls irrespective of the dilution factor (**Figure 2B**). Remarkably, reactivity to phosphoryl adducts was still detectable at 1:3200 - 1:6400 for several subjects, indicating high-titer antibody responses to these adducts. Results were quantified by calculating the area under the curve (AUC) for all titer curves. AUC values were significantly higher in PDAC patients than HD (p-value <0.0001, insets in **Figure 2B**). To determine the subclasses of serum IgG reactive to phosphoryl adducts, we repeated the assay using secondary antibodies specific to IgG1, IgG2, IgG3 and IgG4. Results reported in **Figure 2C** revealed than most of the reactivity could be attributed to IgG1 and IgG3, with minimal signal detected for IgG2 and IgG4.

### IgG responses to phosphoryl adducts and metastases

PDAC is often diagnosed at an advanced stage when the tumor has already spread to distant organs. This situation corresponds to M1 as part of the Tumor, Node, Metastasis (TNM) staging system. By opposition, M0, refers to absence of metastasis beyond regional lymph nodes. To assess whether the presence of metastasis, taken as a surrogate marker of tumor burden, would impact the development of antibodies to phosphoryl adducts, we compared IgG reactivity between patients diagnosed at M0 to those diagnosed at M1 stage. As shown in **Figure 2D**, patients with metastasis appeared to have higher reactivity to these adducts when compared to patients without metastasis, suggesting that higher tumor burden or dissemination may have enhanced antibody responses to these immunogenic targets. In addition to the differences observed between M0 and M1 patients, further analysis of IgG reactivity to phosphoryl adducts revealed significant variations among different patient groups (**Supplementary Figure S3**). Post-treatment PDAC samples (PDAC-PT) exhibited higher IgG levels against phosphorylated adducts compared to healthy controls, suggesting that treatment did not significantly affect antibody responses to these immunogenic targets. Additionally, PDAC patients exhibited higher anti-phosphoryl adduct IgG levels when compared to patients with type 2 diabetes (T2D), suggesting that systemic metabolic alterations associated with type of diabetes, did not influence IgG responses to this modified antigens.

### Serum IgG to phosphoryl-Tyr as a predictor of PDAC

**Figure 3:**
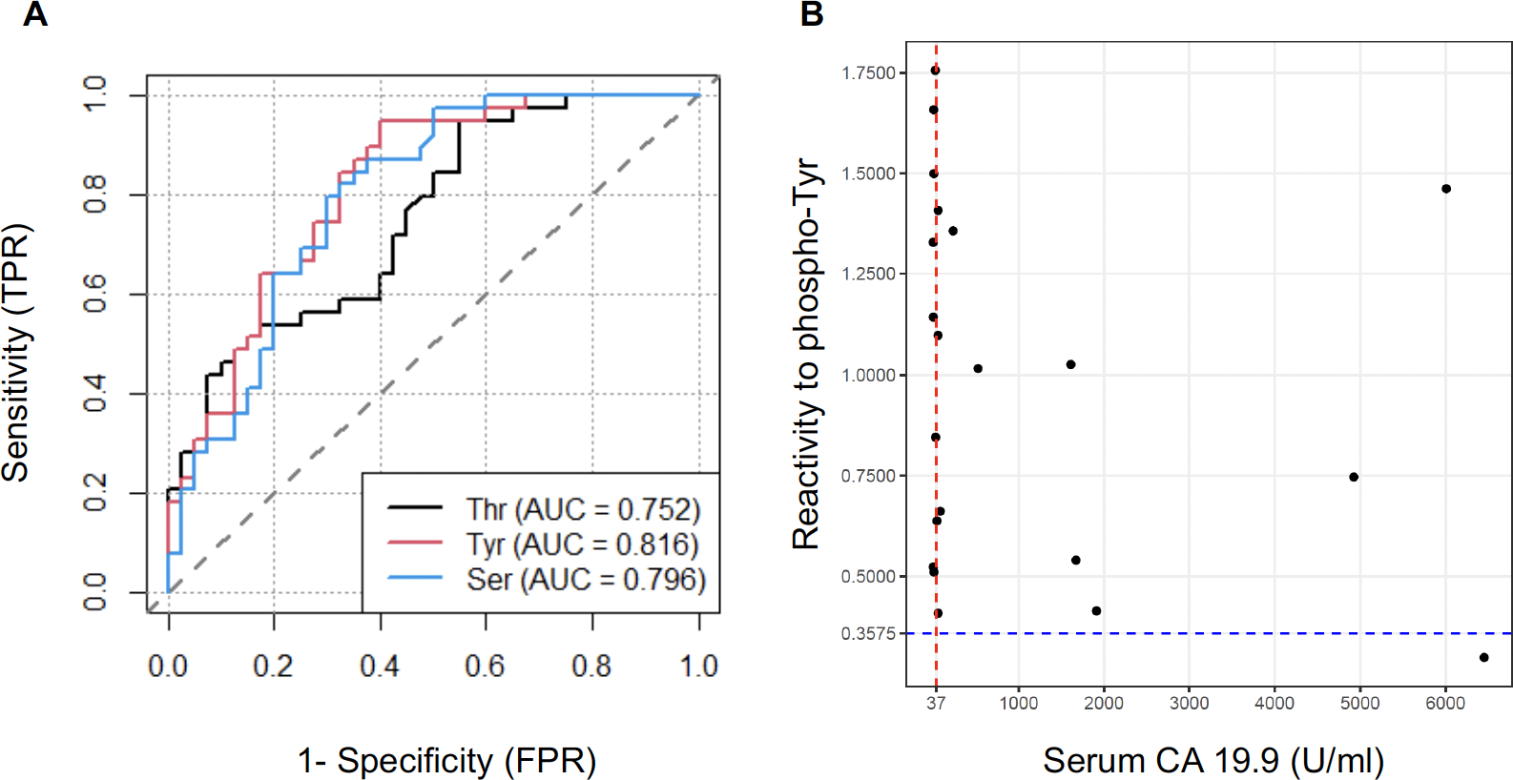
Serum anti-phosphoryl adduct IgG as marker of PDAC. **(A)** Receiver operating characteristic curve (ROC) for discrimination of PDAC from CTRL based on serum IgG reactivity to 3 phosphoryl groups. **(B)** Comparison between serum CA19.9 levels and reactivity to phosphoryl-Tyr as predictive markers for the diagnosis of PDAC. The CA19.9 reference cutoff (37U/ml) is indicated by a red line. The optimal cutoff point for reactivity to phosphoryl-Tyr (0.37) is indicated by a blue dotted line.

We then refined our predictive model to identify which of the 3 variables (i.e., IgG reactivity to phosphoryl-Ser, -Thr or -Tyr) was the most performant to discriminate between PDAC and controls. Moreover, beyond the prediction power of each individual variable, we also evaluated the performance of using a combination of two or three of these markers. As a first metric we calculated the patient-level sensitivity or true positive rate (TPR) defined as the probability of a PDAC case diagnosed. We also calculated the false positive rate (FPR or 1-specificity), defined as the probability of a wrong diagnosis of a control patient as a cancer case. Accuracy (ACC) was defined as the rate of true positive and true negative. Lastly, we calculated the area under the receiver operating curve (AUC-ROC) another critical measurement to quantify the model’s discriminatory power. Sensitivity, specificity, accuracy and AUC were calculated at an optimal cutoff point determined using the Youden index. All metric values for the 3 individual variables as well as all combinations of 2 or 3 variables are reported in **Supplementary Table S4**. **Figure 3A** depicts the ROC for the 3 phosphoryl adducts. Overall, the models were very similar for all 3 variables and combinations thereof with respect to their discriminatory power. IgG reactivity to phosphoryl-Tyr appeared as the most performant variable with a sensitivity of 94.9% and an AUC of 81.6% but was only marginally superior to the two other variables. This observation is consistent with the fact that reactivity to phosphoryl-Ser, -Thr and -Tyr were highly correlated (pairwise Pearson correlation > 0.7).

The Sialyl Lewis antigen A or carbohydrate antigen 19-9 (CA19.9) is currently the only FDA-approved biomarker for the detection and monitoring of PDAC. We compared the predictive value of IgG reactivity to phosphoryl-Tyr to that of CA19.9 in our case-control sample. Of note, CA19.9 values at time of diagnosis were only available for 21/39 patients. **Figure 3B** reports the measurement for the 21 PDAC cases with the standard cutoff point of 37U/ml for CA19.9 and the previously determined cutoff point of 0.375 indicated by doted lines. As illustrated in this figure, IgG reactivity to phosphoryl-Tyr was superior to CA 19.9 in discriminating PDAC patients. Comparison between CA19.9 levels and IgG reactivity to phosphoryl-Ser and -Thr are reported in **Supplementary Figure S4**.

### Higher levels of phosphoryl adducts in PDAC cells

**Figure 4:**
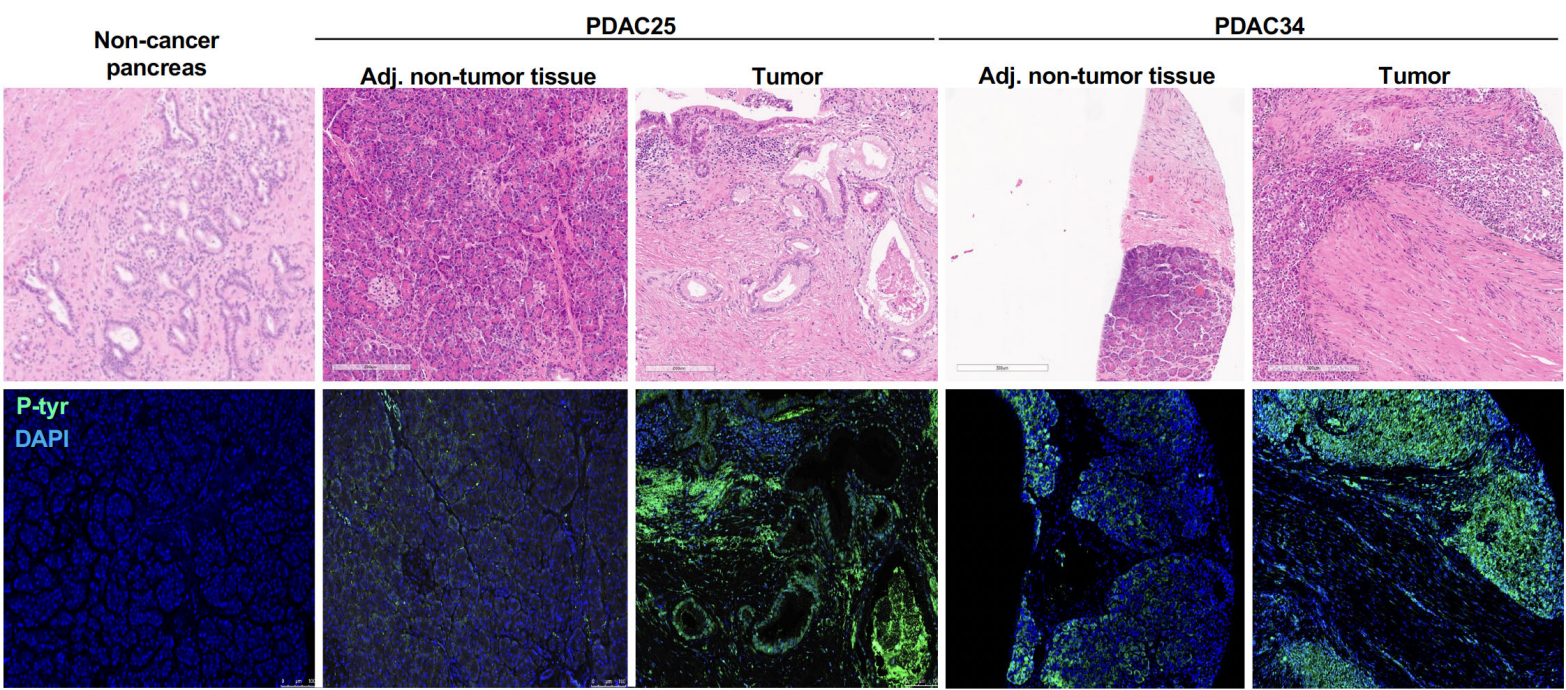
Accumulation phosphoryl adducts in tumor cells. Immunofluorescence staining of tumor tissue and adjacent non-tumoral tissue for two representative PDAC cases as well as control non-cancerous pancreatic tissue with an anti-phosphoryl-Tyr mab (green) and DAPI (blue). Staining of consecutive tissue sections with hematoxylin and eosin (H&E) is also shown.

To elicit high titer class-switched antibody responses and leave a serum “IgG footprint”, we reasoned that these adducts would have to be aberrantly expressed in tumor cells. To test this hypothesis, we assessed the presence of phosphoryl-Tyr directly in pancreatic tumor tissue. Detection of phosphoryl-tyrosine by immunofluorescence was performed in 13 cases of PDAC who had previously demonstrated elevated anti-phosphoryl-Tyr IgG levels and for whom formalin-fixed paraffin embedded tissue was available. For 11 cases, adjacent non-tumoral tissue was also available and stained as control. **Figure 4**, reports two representative example showing much higher levels of phosphoryl-Tyr in the tumor area when compared to the adjacent non-tumoral area or non-cancerous pancreatic tissue. The remaining cases are reported in **Supplementary Figure S5**. A summary of staining results is provided in **Table 1**. On the whole, aberrant buildup of phosphoryl-Tyr in tumor cells was unmistakably observed in 13 out of 13 PDAC patients. In contrast, adjacent non-tumoral tissue either did not stain for phosphoryl-Tyr (6/11 cases) or showed minimal staining of acinar cells, benign duct cells, endothelial cells or infiltrating lymphocytes (5/11 cases).

**Table 1:** Detection of phosphorylated tyrosine in tumor and adjacent non-tumoral tissue by immunofluorescence.

Study code	Phosphoryl-tyrosine staining		
	Tumor tissue	Adjacent non-tumoral tissue	
PDAC6	+	–	
PDAC7	+	–	
PDAC8	++	+	Acinar cells
PDAC16	++	+	Bening ducts​
PDAC21	+++	+	Endothelial cells
PDAC25	+	–	
PDAC27	+++	–	
PDAC28	+++	NA	
PDAC29	+++	-	
PDAC34	+++	+	Lymphocytes​
PDAC37	+++	–	
PDAC38	+++	NA	
PDAC39	+++	++	Bening ducts

### Phosphoproteome analysis of PDAC

**Figure 5:**
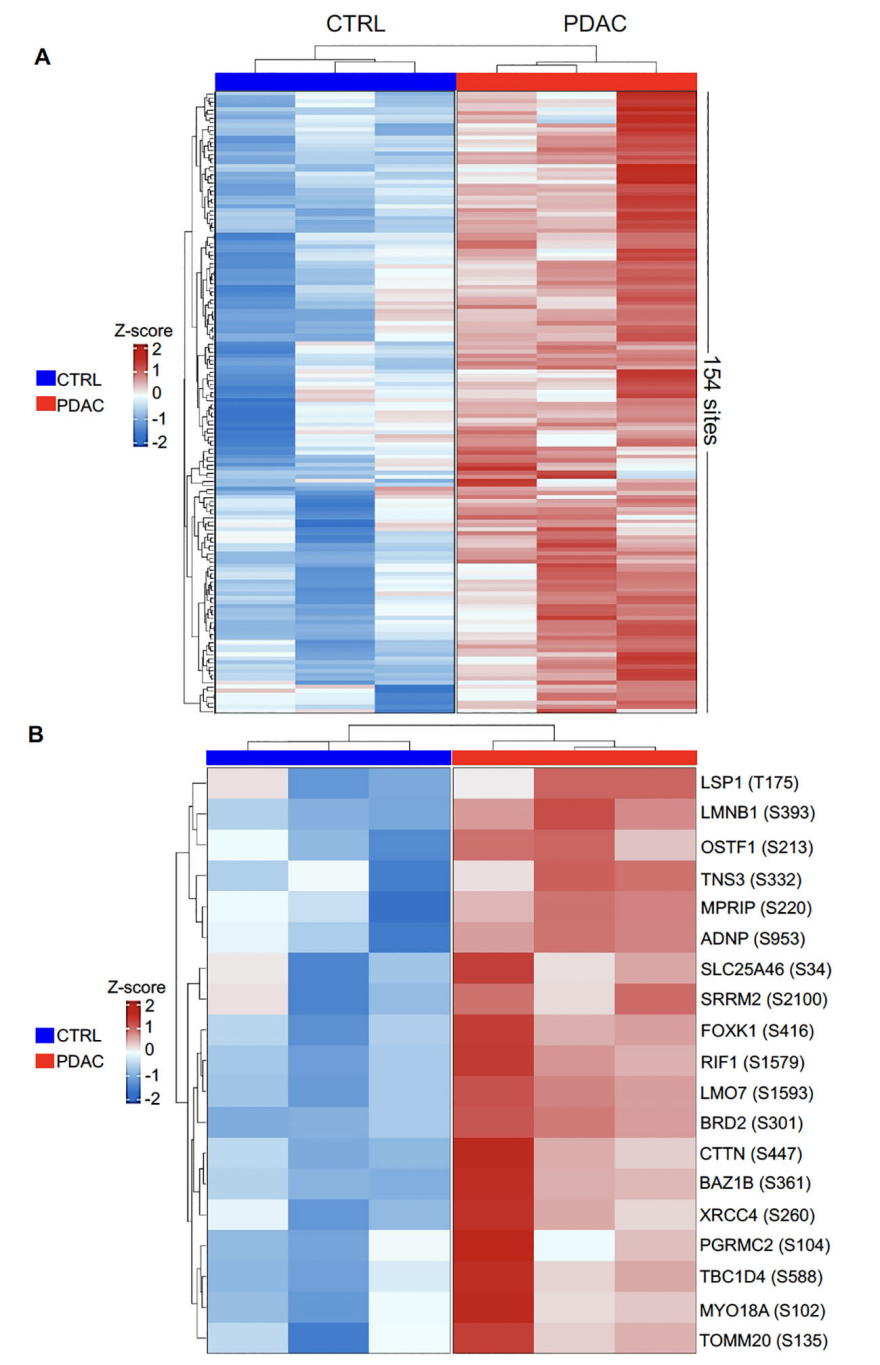
Heatmap representation of differentially phosphorylated sites between PDAC and CTRL. Normalized values were scaled to convert to z-scores. **(A)**. 154 differentially phosphorylated sites corresponding to 124 proteins, were identified at p< 0.05 with logFC greater than 1; **(B)** 19 differentially phosphorylated sites were identified at p< 0.01 with logFC greater than 1.

The accumulation of phosphoryl adducts in PDAC cells revealed by immunofluorescence suggested that this cancer type may be associated with dysregulation and abnormal activation of phosphorylation pathways. We next used a mass spectrometry-based phosphoproteomics approach to analyze the phosphoproteome in 3 PDAC patients for whom paired flash-frozen PDAC and adjacent non-tumoral tissue were available. All 3 patients had previously showed elevated serum IgG levels to phosphoryl-adducts. Differential Phosphorylation was computed using the msqrob2 package as specified in the materials and methods section. A total of 10,986 sites were identified as differentially phosphorylated between tumor and non-tumoral cells in any of the 3 samples. Among these, 154 sites corresponding to 124 proteins, showed higher phosphorylation levels in tumor cells compared to adjacent non-tumoral cells in all 3 cases at a statistical significance level of 5% (p<0.05, **Figure 5A**; **Supplementary Table S5**). Nineteen were confirmed at a statistical significance level of 1% (p<0.01, **Figure 5B**; **Supplementary Table S6**).

A gene ontology (GO) enrichment analysis was then carried out to further characterize the 124 proteins identified as aberrantly over-phosphorylated in tumor tissue compared to adjacent non-tumoral pancreatic tissue. This analysis identified 104 biological processes, 18 cellular components and 10 molecular functions listed in **Supplementary Table S7**. A visual summary of this GO analysis is presented in **Supplementary Figure S6**. Key biological processes include protein localization to chromosomes, regulation of transcription by RNA polymerase I, and regulation of histone acetylation (**Supplementary Figure S6A**). Enriched cellular components indicated involvement in nuclear protein-containing complexes, pericentromeric heterochromatin, and ribonucleoprotein complexes (**Supplementary Figure S6B**). Lastly, molecular functions highlighted chromatin binding, molecular adaptor activity, and RNA binding, suggesting a role in chromatin organization and gene expression regulation (**Supplementary Figure S6C**).

## Discussion and conclusion

Antibody responses to solid tumors have been described more than 20 years ago. The original work by the group of Pfreundschuh and Sahin identified a series of melanoma- antigens through the SEREX method (5). Since this early report, numerous studies have investigated serological responses to various cancer types, including PDAC and identified a large array of tumor associated antigens (TAA) (3, 23). These included several cancer-testis antigens but most predominantly unmutated self-proteins, some of which were overexpressed in cancer cells. Another category of TAA appears to draw its immunogenicity from post-translational modification (PTM). For instance, serum antibodies to phosphorylated α-Enolase were reported in 2011 as a common characteristic of PDAC (24). In this situation, IgG only bound the phosphorylated form of the antigen, indicating that the modification was essential to its recognition. More recently, PTM-driven TAA was also described in the context of non-small cell lung cancer (NSCLC) (25). Following the same reasoning, we used a unique panel developed in our lab (22) to examine the presence of IgG in a wide range of modifications in the serum of PDAC patients. Among the recognized adducts, phosphoryl groups on serine, tyrosine, and threonine were the dominant targets in line with the previous report on phosphorylated α-Enolase.

Carcinogenesis of PDAC is associated with multiple cell modifications including abnormal ubiquitination, SUMOylation, sialylation, N-linked-glycosylation and other PTM (16, 18–20). Profound perturbations of the phosphoproteome were among the most significant transformations observed in PDAC as evidenced in a comprehensive proteogenomic analysis (26). Upregulation of several kinases, such as PAK1 and PAK2, largely contribute to both excess and aberrant phosphorylation patterns in transformed cells. Our studies corroborated these findings and identified a series of proteins differentially phosphorylated between tumor and normal cells. The 5 most important are 1) Lineage-specific Interacting Motif domain only 7 (LMO7), this protein plays a role in cell adhesion and cytoskeletal organization. Increased phosphorylation may affect cell migration and invasion, contributing to tumor progression (27); 2) Lamin B1 (LMNB1), this B-type lamin protein, is a nuclear lamina component involved in nuclear structure and gene regulation. Its excess phosphorylation may impact nuclear integrity and abnormal gene expression, potentially influencing tumor growth and metastasis (28); 3) Progesterone Receptor Membrane Component 2 (PGRMC2) has been attributed key roles in tumor growth (29, 30); 4) Bromodomain-containing protein 2 (BRD2) it is a transcriptional regulator involved in chromatin remodeling and transcriptional regulation. BRD2 was involved in PDAC progression through the control of hyaluronidase 1 (31). Increased phosphorylation BRD2 may affect gene expression patterns and influence tumor development, and 5) Myosin18A (MYO18A) this protein is part of a complex that assembles lamellar actomyosin bundles and may be required for migration and invasiveness of cancer cells (32).

Additionally, enriching phosphorylation sites in chromatin organization and transcriptional regulation proteins indicates that dysregulated phosphorylation may contribute to epigenetic modifications and altered gene expression in PDAC (33, 34). Furthermore, the aberrant phosphorylation landscape observed in our cohort likely reflects broader dysregulation of oncogenic pathways, including KRAS-driven signaling cascades, which are known to be overexpressed in PDAC (14, 16, 20, 24, 26). Overall, our phosphoproteome analysis reveals aberrant phosphorylation events most probably linked to the malignant transformation of PDAC cells or their capacity to disseminate. Further investigation should integrate phosphoproteomics with transcriptomic and epigenomic analyses, which will be crucial in defining the functional impact of these modifications on tumor biology. On the other hand, our findings also support the view that aberrantly phosphorylated proteins in PDAC cells act as a potent source of tumor-associated antigens capable of eliciting immune responses. Further, isotype-switched IgG responses to phosphoryl adducts strongly suggest these PTMs may act as neoantigen-like structures, triggering adaptive immunity. Given the robustness of these antibody responses, we propose that phosphorylated self-proteins significantly contribute to the tumor’s immunogenic landscape. This hypothesis aligns with recent reports of B cell infiltrates in PDAC and suggests a broader role for PTM-driven antigenicity in shaping tumor-immune interactions.

Finding higher levels of phosphoryl-Tyr in the tumor of patients with elevated serum reactivity to this PTM substantiated our main hypothesis that B cell responses develop to chemical adducts accumulating in cancer cells. All specimens were collected from PDAC patients before chemotherapy, underscoring the spontaneous nature of anti-adduct B cell responses. While our cross-sectional study does reveal the timing of these responses, the high antibody titers including primarily isotype-switched IgG1 and IgG3 observed for most cases strongly suggest that these developed long before the cancer diagnosis. The higher serum reactivity to phosphoryl adducts seen in patients with metastatic PDAC compared to non-metastatic cases, also points to a possible association between tumor burden and the robustness of the immune response. Longitudinal prospective studies using serial samples collected at time intervals before diagnosis with detailed epidemiologic and clinical data are now warranted to confirm this view. Additionally, we assessed samples collected from patients, such as those with type 2 diabetes (T2D). Results suggested that systemic metabolic alterations associated with T2D, did not trigger IgG responses to phosphoryl adducts.

Our report focuses predominantly on phosphoryl-tyrosine, -threonine or -serine. However, our initial screening identified 10 additional adduct targets using a classifier method combined with the Boruta algorithm. Following our hypothesis, these modifications could also be abundant in cancer cells. More generally, we posit that specific antibody signatures detected in PDAC patients are likely to reflect the immunogenic adductome of the corresponding tumor cells.

Immunoprofiling using protein microarrays characterized specific autoantibody signatures with potential value as diagnostic tools in PDAC (12–15). The anti-adduct signature reported here could also be utilized as a biomarker for this cancer type. In this regard, it is noteworthy that IgG reactivity to phosphoryl group on any of the 3 amino acids outperformed serum levels of CA19.9 to discriminate between cases and controls. As mentioned above, additional studies will determine if anti-phosphoryl adduct IgG can also be detected months or years before PDAC becomes symptomatic and serve as an early, non-invasive marker for this deadly disease.

B cell and plasma cell infiltrates are common in PDAC (35, 36). A recent study by Dr. Fearon and colleagues reports that these plasma cells included clones secreting antibodies reactive to self-antigens such as filamentous actin (37). Based on our data, it is plausible that a fraction of infiltrating B cells and plasma cells also target immunogenic PTM accumulating in the cancer cells, e.g., phosphoryl adducts as well as modified self-antigens exposing these adducts. This hypothesis, if verified, would establish a link between intratumor B cell immunity and serological responses. In addition, the presence of high-titer IgG responses to phosphorylated proteins in PDAC patients strongly suggests the involvement of phosphorylated adduct-specific T helper cells, providing the necessary help for B cells to undergo class switching from IgM to IgG through a coordinated immune response (24).

In conclusion, the present study reports the first comprehensive characterization of anti-adduct serological responses in PDAC. Results unequivocally identified phosphoryl moieties as shared tumor-associated adducts, with most patients displaying isotype-switched high-titer antibodies responses to these PTM. Our findings provide a stepping stone towards understanding natural B cell immunity to PDAC and developing therapeutic antibodies to target this cancer.

## Data Availability

The data presented in the study are deposited in the PeptideAtlas repository, accession number PASS05908/(http://www.peptideatlas.org/PASS/PASS05908).

## Supplementary material

Supplementary Figure 1 Differential IgG reactivity to the 13 most relevant adducts identified by random forest classifier and Boruta analysis between PDAC (red) and CTRL (blue)}. ****p-value < 0.0001; ***p-value < 0.001 (Unpaired t test with Welch’s correction).

Supplementary Figure 2 Principal component analysis (PCA) of PDAC patients (red) and CTRL (blue) IgG reactivity to 93 adducts between.

Supplementary Figure 3 Anti-phosphorylated adduct IgG levels in the serum of patients with type 2 diabetes and PDAC post-treatment samples. Differential IgG reactivity to the 3 phosphorylated amino acids between controls **(A)** (CTRL, blue, n=40) and PDAC – PT (post-treatment samples, dark red, n=12) - phosphoryl threonine p-value = 0.0014; phosphoryl serine p-value = 0.0873; phosphoryl tyrosine p-value = 0.0145. **(B)** PDAC (red n=39) and DM2 (Diabetes type 2 samples, grey n=30) - phosphoryl threonine p-value <0.0001; phosphoryl serine p-value <0.0001; phosphoryl tyrosine p-value = 0.0002. (Unpaired t-test with Welch’s correction).

Supplementary Figure 4 Comparison between serum CA19.9 levels and reactivity to phosphoryl-Thr and -Ser as predictive markers for the diagnosis of PDAC. The CA19.9 reference cutoff (37U/ml) is indicated by a red line. The optimal cutoff points for reactivity to phosphoryl-Thr and -Ser are indicated by doted lines.

Supplementary Figure 5 Detailed immunofluorescence of the detection of phosphoryl-tyrosine in tissue samples tested.

Supplementary Figure 6 Functional landscape of aberrantly phosphorylated proteins in PDAC revealed through Gene Ontology analysis**. (A)** Dot plot of main biological processes. **(B)** CNETplot of cellular components. **(C)** CNETplot of molecular functions.
