# Supplementary figures and images for: Development of high-titer class-switched antibody responses to phosphorylated amino acids is prevalent in pancreatic ductal adenocarcinoma
Source: Front Immunol. 2025 Mar 28;16:1501943. doi: 10.3389/fimmu.2025.1501943 (PMC11985851; doi:10.3389/fimmu.2025.1501943)

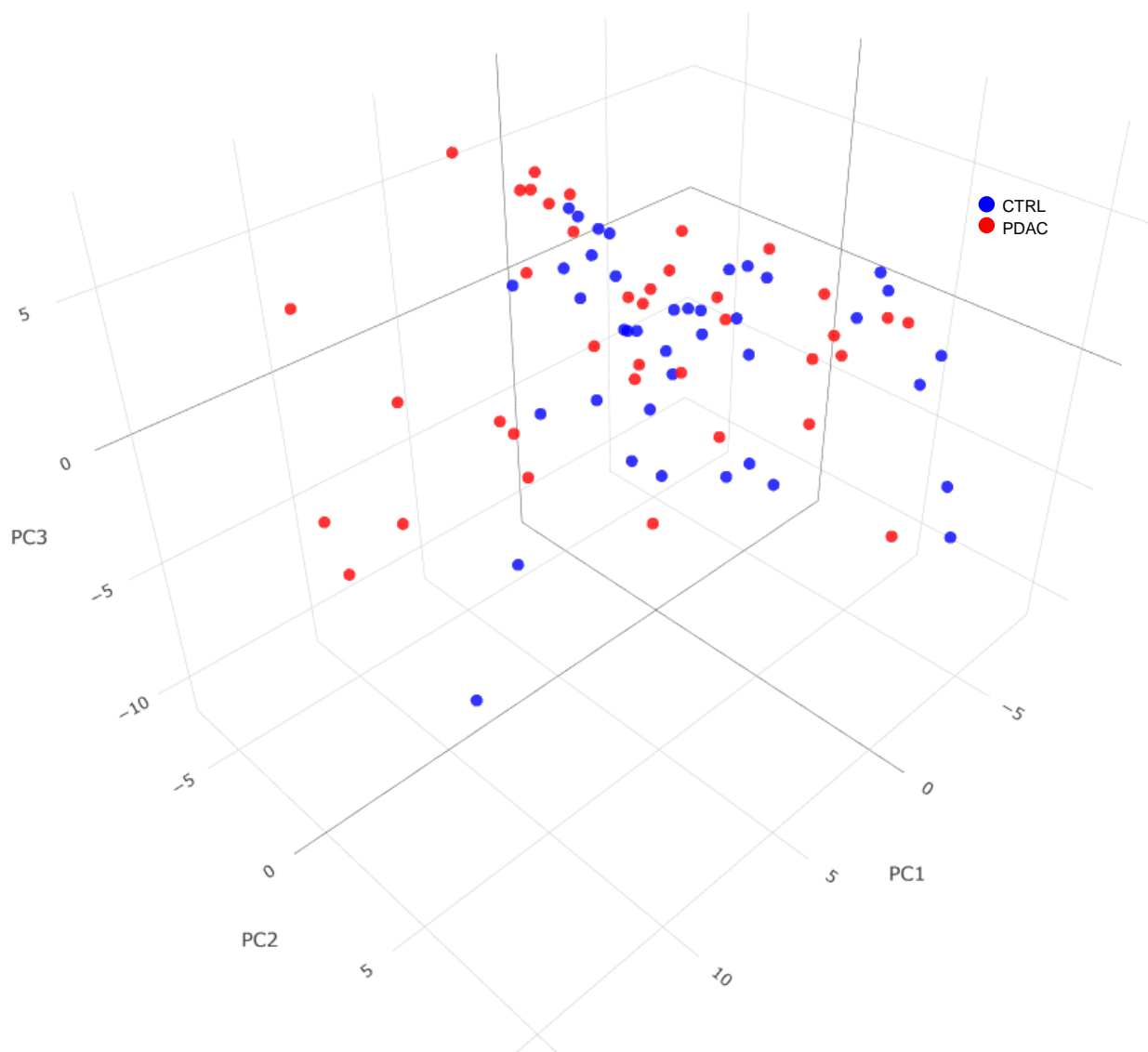

Supp. Figure S2

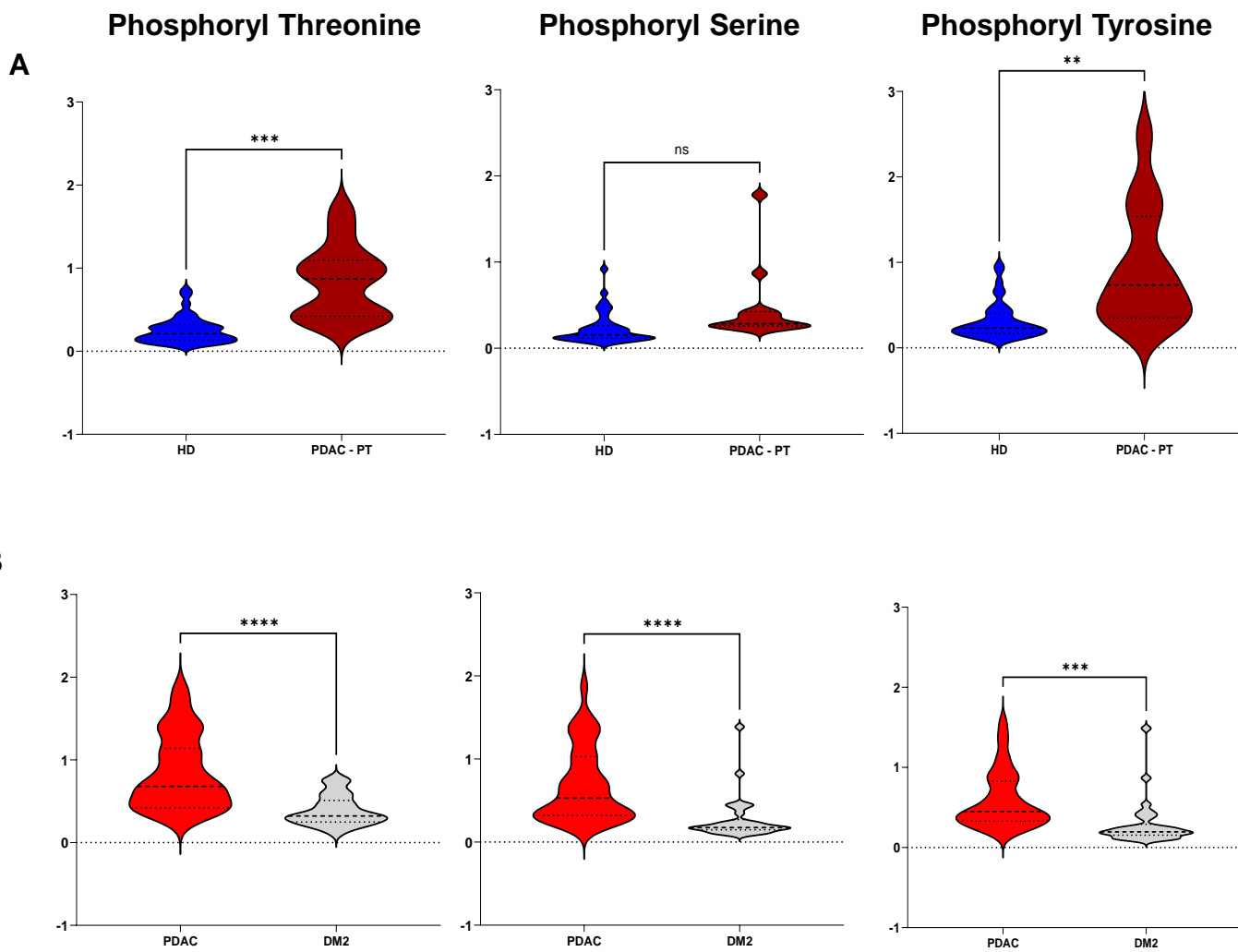

Supp. Figure S3

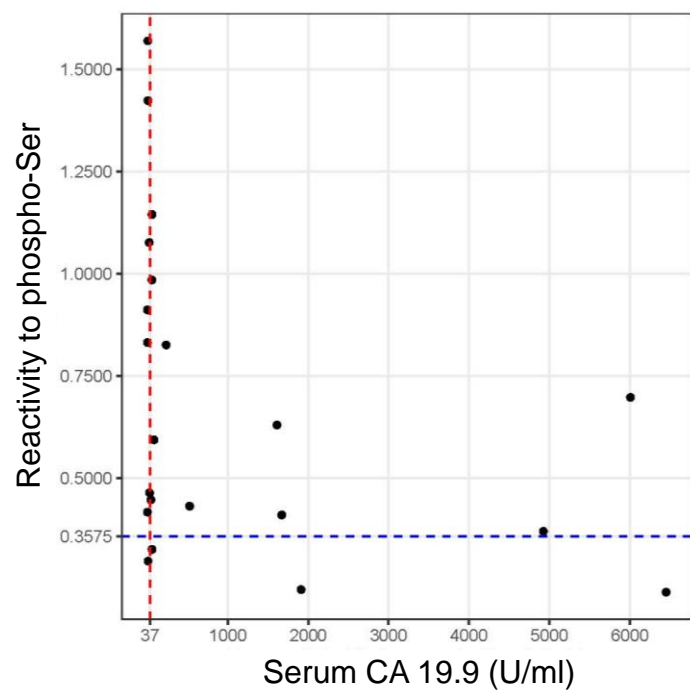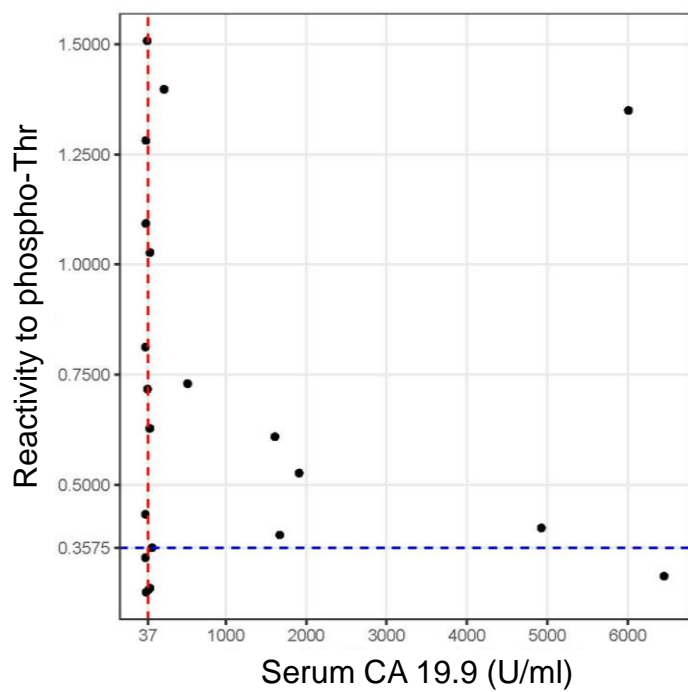

Supp. Figure S4

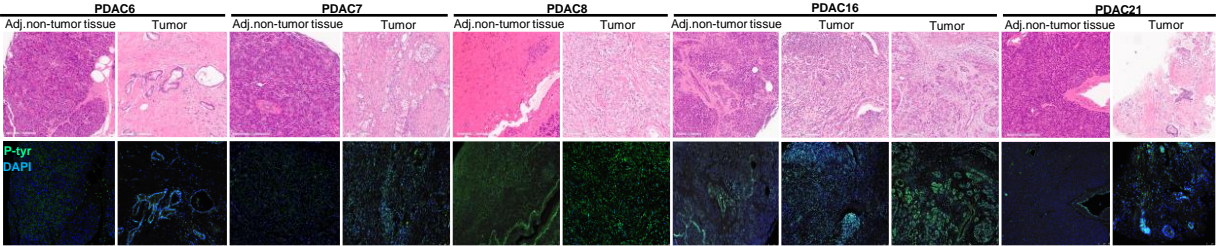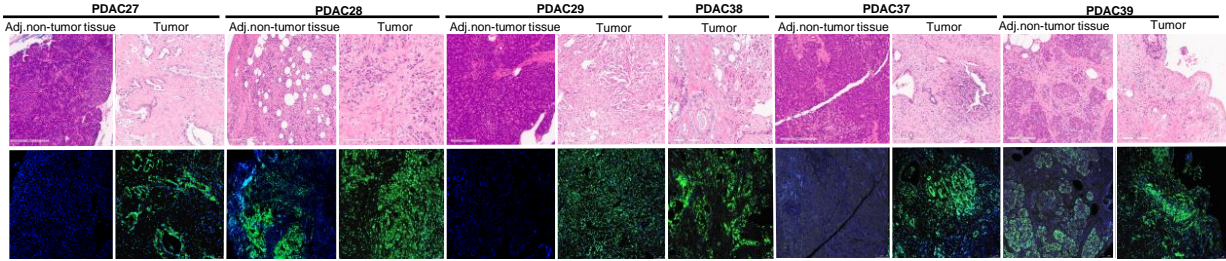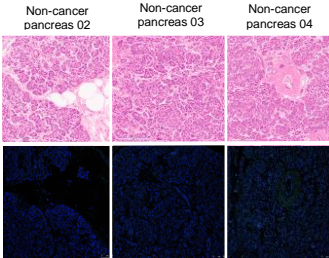

Supp. Figure S5

**A**

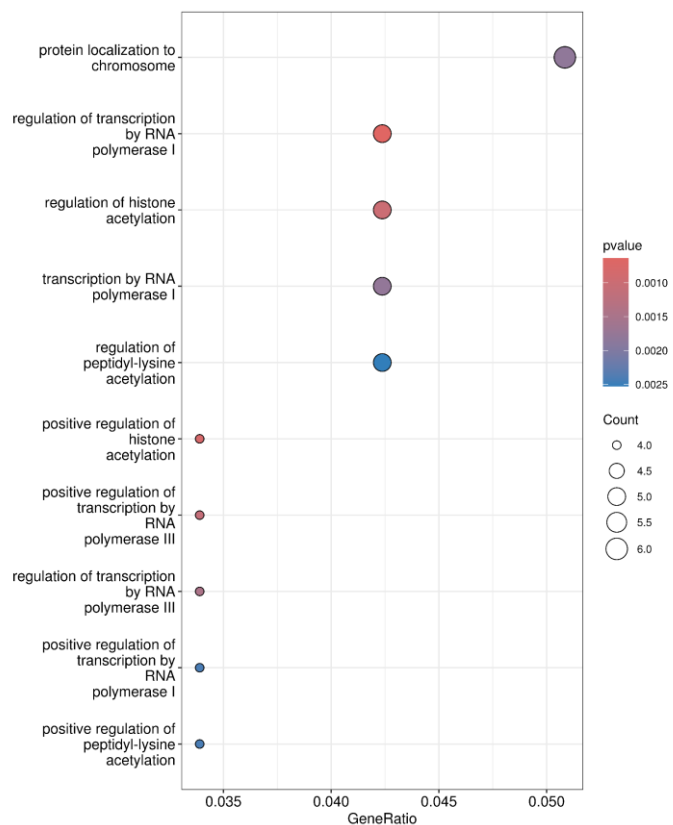

**B**

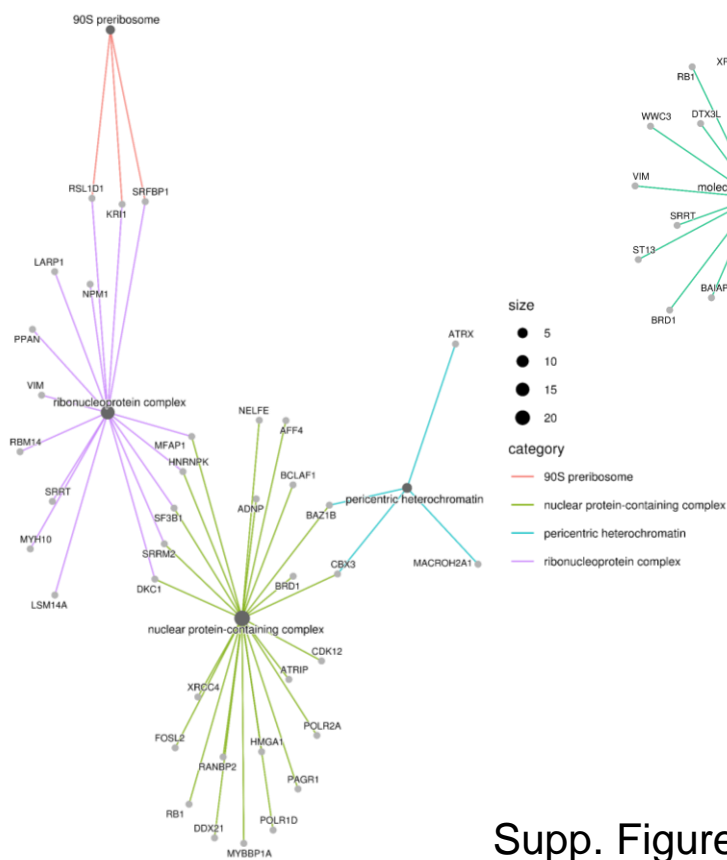

**C**

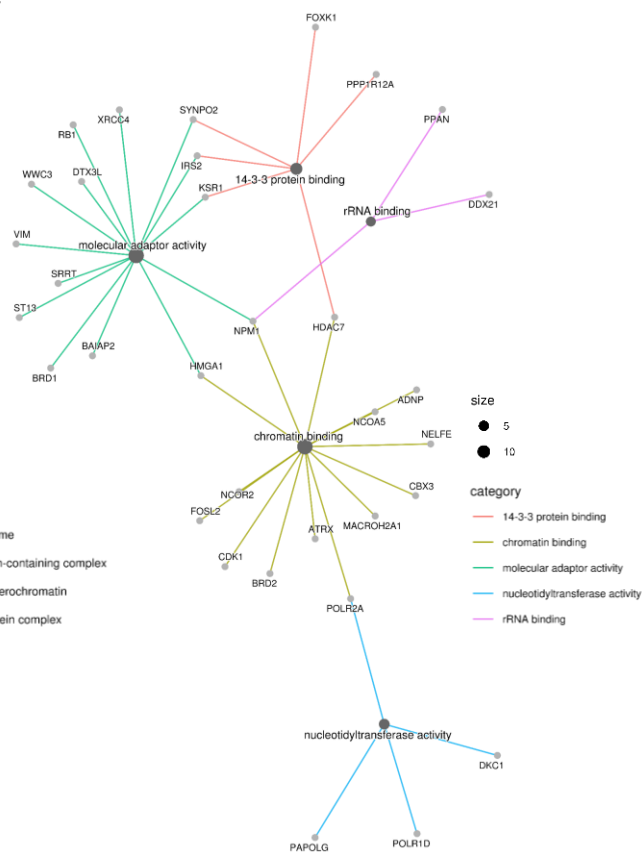

Supp. Figure S6

Supplement: Supplementary Figure 1 — Differential IgG reactivity to the 13 most relevant adducts identified by random forest classifier and Boruta analysis between PDAC (red) and CTRL (blue)}. ****p-value < 0.0001; ***p-value < 0.001 (Unpaired t test with Welch’s correction). [file DataSheet1.pdf]
